# Supplementary material for: The single-cell transcriptomic atlas and RORA-mediated 3D epigenomic remodeling in driving corneal epithelial differentiation
Source: Nat Commun. 2024 Jan 4;15:256. doi: 10.1038/s41467-023-44471-w (PMC10766623; doi:10.1038/s41467-023-44471-w)
Supplement: Supplementary file 1 — Supplementary Information [file 41467_2023_44471_MOESM1_ESM.pdf]

Supplementary Information for

The single-cell transcriptomic atlas and RORA-mediated 3D epigenomic remodeling in driving corneal epithelial differentiation

Mingsen Li[1,*†], Huizhen Guo[1†], Bofeng Wang[1†], Zhuo Han[1], Siqi Wu[1], Jiafeng Liu[1], Huaxing Huang[1], Jin Zhu[1], Fengjiao An[1], Zesong Lin[1], Kunlun Mo[1], Jieying Tan[1], Chunqiao Liu[1], Li Wang[1], Xin Deng[2], Guigang Li[3], Jianping Ji[1,*], Hong Ouyang[1,*]

*Corresponding: Ouyhong3@mail.sysu.edu.cn; lims3@mail2.sysu.edu.cn; jpji1974@126.com.

†These authors contributed equally to this work.

This PDF file includes:

Figs. S1 to S4

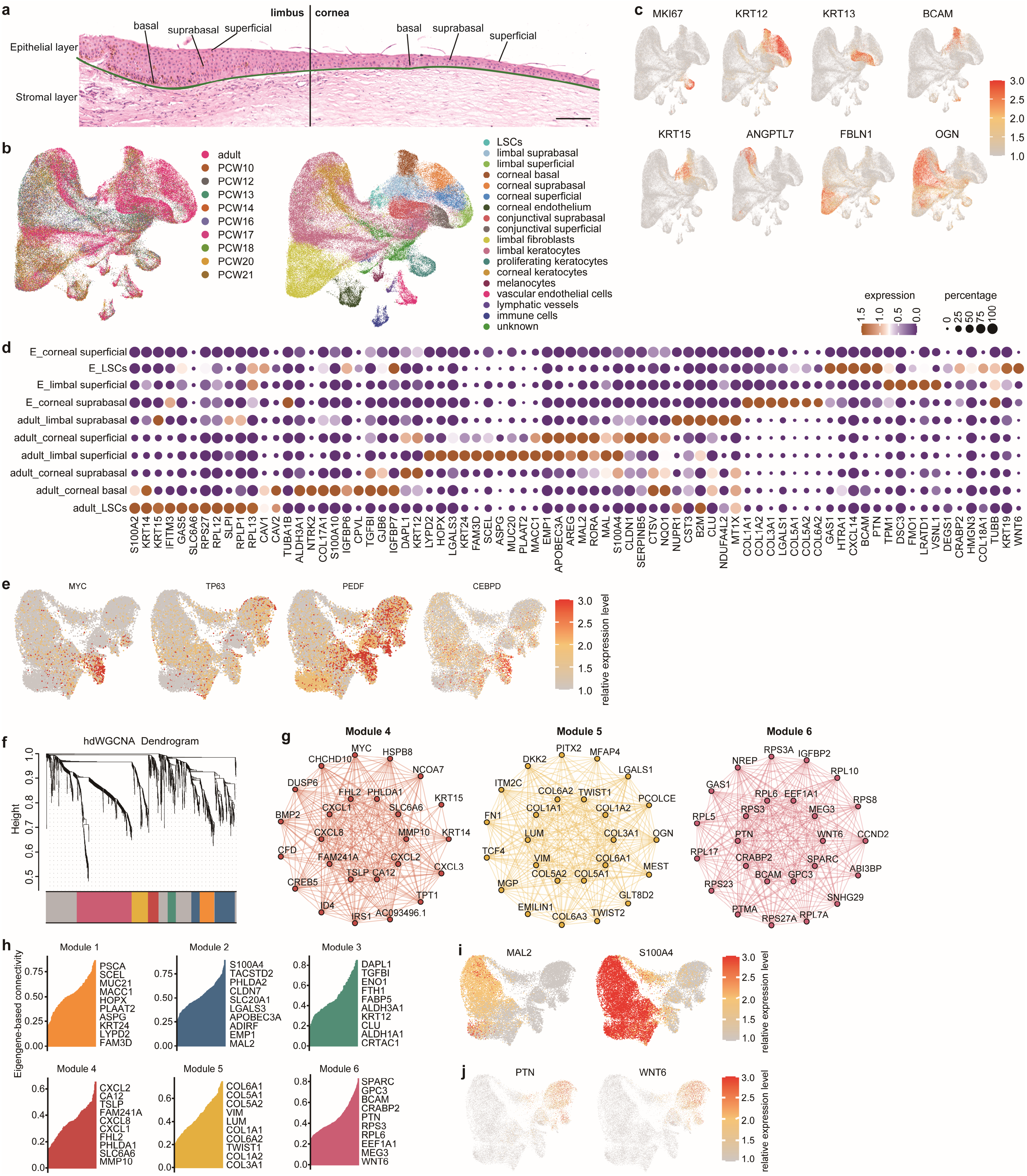
a
limbus
cornea
basal
suprabasal
superficial
Epithelial layer
Stromal layer
b
adult
PCW10
PCW12
PCW13
PCW14
PCW16
PCW17
PCW18
PCW20
PCW21
LSCs
limbal suprabasal
limbal superficial
corneal basal
corneal suprabasal
corneal superficial
corneal endothelium
conjunctival suprabasal
conjunctival superficial
limbal fibroblasts
limbal keratocytes
proliferating keratocytes
corneal keratocytes
melanocytes
vascular endothelial cells
lymphatic vessels
immune cells
unknown
c
MKI67
KRT12
KRT13
BCAM
KRT15
ANGPTL7
FBLN1
OGN
3.0
2.5
2.0
1.5
1.0
expression
percentage
1.5 1.0 0.5 0.0
0 25 50 75 100
d
E_corneal superficial
E_LSCs
E_limbal superficial
E_corneal suprabasal
adult_limbal suprabasal
adult_corneal superficial
adult_limbal superficial
adult_corneal suprabasal
adult_corneal basal
adult_LSCs
S100A2 KRT14 KRT15 IFITM3 GAS5 SLC6A6 RPS27 RPL12 SLPI RPLP1 RPL13 CAV1 CAV2 TUBA1B ALDH3A1 NTRK2 COL17A1 S100A10 IGFBP6 CPVL TGFBI GJB6 IGFBP7 DAPL1 KRT12 LYPD2 HOPX LGALS3 KRT24 FAM3D SCEL ASPG MUC20 PLAAT2 MACC1 EMP1 APOBEC3A AREG MAL2 RORA MAL S100A4 CLDN1 SERPINB5 CTSV NQO1 NUPR1 CST3 B2M CLU NDUFA4L2 MT1X COL1A1 COL1A2 COL3A1 LGALS1 COL5A1 COL5A2 COL6A2 GAS1 HTRA1 CXCL14 BCAM PTN TPM1 DSC3 FMO1 LRATD1 VSNL1 DEGS1 CRABP2 HMGN3 COL18A1 TUBB KRT19 WNT6
e
MYC
TP63
PEDF
CEBPD
relative expression level
f
hdWGCNA Dendrogram
Height
1.0 0.9 0.8 0.7 0.6 0.5
g
Module 4
MYC
HSPB8
CHCHD10
NCOA7
DUSP6
FHL2
PHLDA1
KRT15
CXCL1
SLC6A6
BMP2
CXCL8
MMP10
KRT14
CFD
FAM241A
CXCL2
CXCL3
TSLP
CA12
CREB5
TPT1
ID4
IRS1
AC093496.1
Module 5
PITX2
DKK2
MFAP4
LGALS1
ITM2C
COL6A2
TWIST1
PCOLCE
COL1A1
COL1A2
FN1
LUM
COL3A1
OGN
TCF4
VIM
COL6A1
MEST
COL5A2
COL5A1
MGP
GLT8D2
EMILIN1
COL6A3
TWIST2
Module 6
RPS3A
IGFBP2
NREP
RPL10
GAS1
RPL6
EEF1A1
RPS8
RPS3
MEG3
RPL5
PTN
WNT6
CCND2
RPL17
CRABP2
SPARC
BCAM
GPC3
ABI3BP
RPS23
SNHG29
PTMA
RPS27A
RPL7A
h
Eigengene-based connectivity
Module 1
PSCA
SCEL
MUC21
MACC1
HOPX
PLAAT2
ASPG
KRT24
LYPD2
FAM3D
Module 2
S100A4
TACSTD2
PHLDA2
CLDN7
SLC20A1
LGALS3
APOBEC3A
ADIRF
EMP1
MAL2
Module 3
DAPL1
TGFBI
ENO1
FTH1
FABP5
ALDH3A1
KRT12
CLU
ALDH1A1
CRTAC1
Module 4
CXCL2
CA12
TSLP
FAM241A
CXCL8
CXCL1
FHL2
PHLDA1
SLC6A6
MMP10
Module 5
COL6A1
COL5A1
COL5A2
VIM
LUM
COL1A1
COL6A2
TWIST1
COL1A2
COL3A1
Module 6
SPARC
GPC3
BCAM
CRABP2
PTN
RPS3
RPL6
EEF1A1
MEG3
WNT6
i
MAL2
S100A4
j
PTN
WNT6

Supplementary Fig. 1 A single-cell atlas of the ocular surface tissue. a H&E staining showing the morphology and structure of the limbus–cornea tissue. Scale bar, 100 μm. **b** UMAP plots showing cell clusters of the human ocular surface tissues from the indicated human embryonic and adult samples. **c** UMAP plots showing the expression of the indicated marker genes. **d** Dot plot showing the top markers for the indicated cell types. **e** UMAP plots showing the expression of *MYC*, *TP63*, *PEDF* and *CEBPD*. **f** hdWGCNA dendrogram showing the identified co-expression modules. **g** hdWGCNA module network plots showing the top 25 genes by the eigengene-based connectivity for module 4, 5, 6. Each edge represents the co-expression relationship between two genes in the network. The top 10 hub genes are placed in the center of the plot, while the remaining 15 genes are placed in the outer circle. **h** The top 10 genes in each module ranked by eigengene-based connectivity. **i, j** UMAP plots showing the expression of *MAL2*, *S100A4*, *PTN* and *WNT6*.

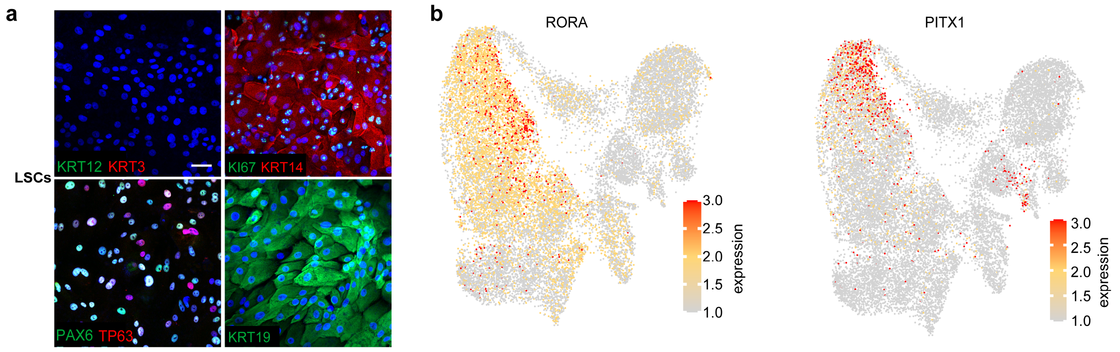

Supplementary Fig. 2 The gene expression of LSCs and the limbal–corneal epithelium. a Immunofluorescence staining for the indicated genes in human primary LSCs. Scale bar, 100 μm. n = 3 biologically independent experiments. **b** UMAP plots showing the expression of *RORA* and *PITX1*.

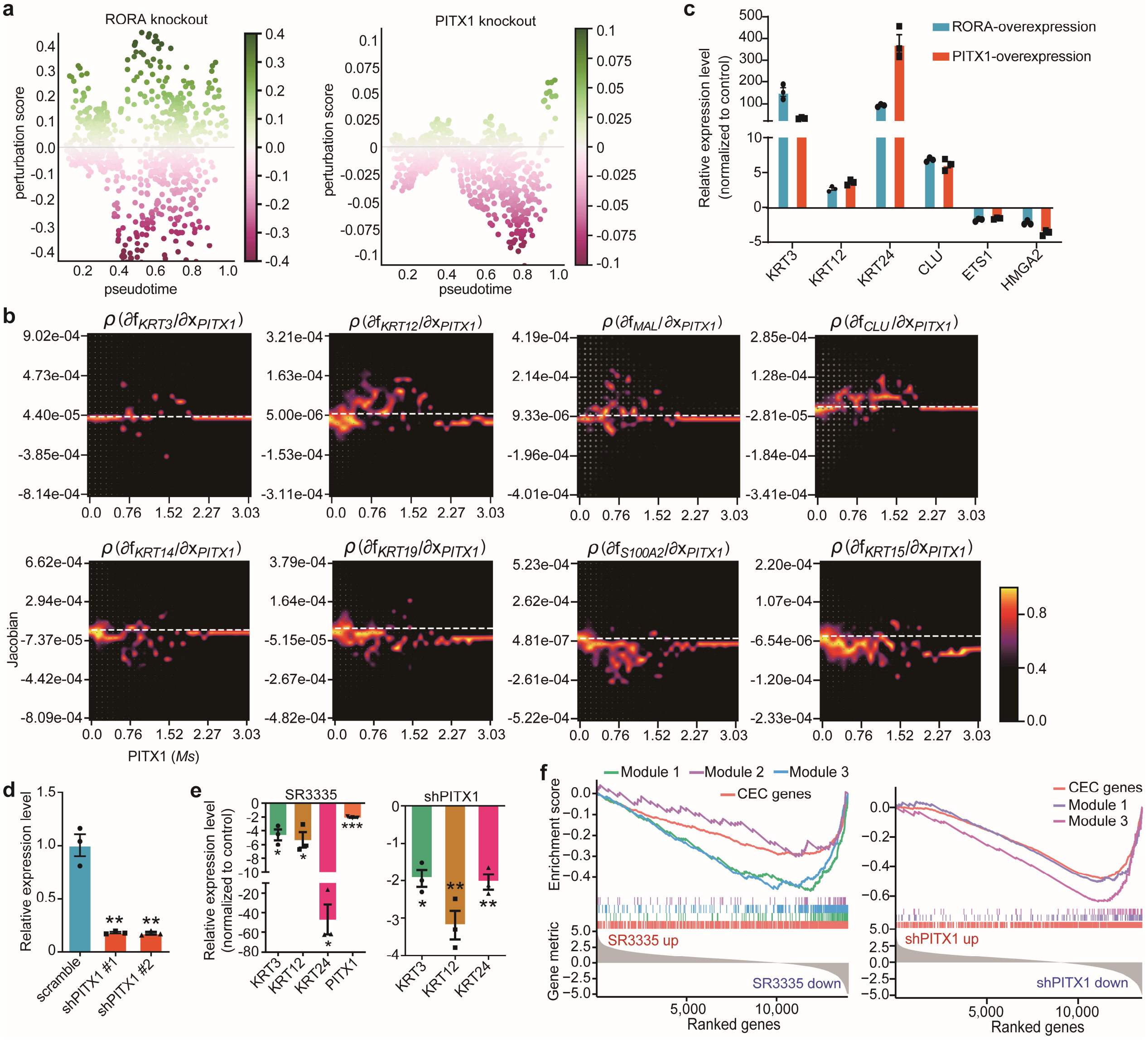

Supplementary Fig. 3 RORA and PITX1 are required for CEC differentiation. a CellOracle *in silico* perturbation scores of *RORA* and *PITX1* in pseudotime. **b** Dynamo response heatmaps showing Jacobian regulation from *PITX1* to the selected genes versus *PITX1 Ms*. White dashed lines indicate the zero value. Positive value represents activation from *PITX1* to the indicated genes and negative represents repression from *PITX1* to the indicated genes. **c** q-RT PCR analysis for the indicated gene expression changes induced by *RORA* and *PITX1* overexpression. Data are represented as means ± SEM (n = 3 independent experiments). Source data are

provided as a Source Data file. **d** q-RT PCR analysis for the expression of *PITX1* in scrambled shRNA- and *shPITX1*-treated CECs. Data are represented as means ± SEM (n = 3 independent experiments, **P < 0.01). *P*-values were calculated using two-sided unpaired Student's t tests. Source data are provided as a Source Data file. **e** q-RT PCR analysis for expression of the indicated genes in the induced CECs treated with SR3335 and *shPITX1* for seven days upon differentiation. Data are represented as means ± SEM (n = 3 independent experiments, **P* < 0.05, ***P* < 0.01, ****P* < 0.001). *P*-values were calculated using two-sided unpaired Student's t tests. Source data are provided as a Source Data file. **f** GSEA for the indicated gene sets using bulk RNA-seq data of SR3335- and *shPITX1*- versus control-treated CECs.

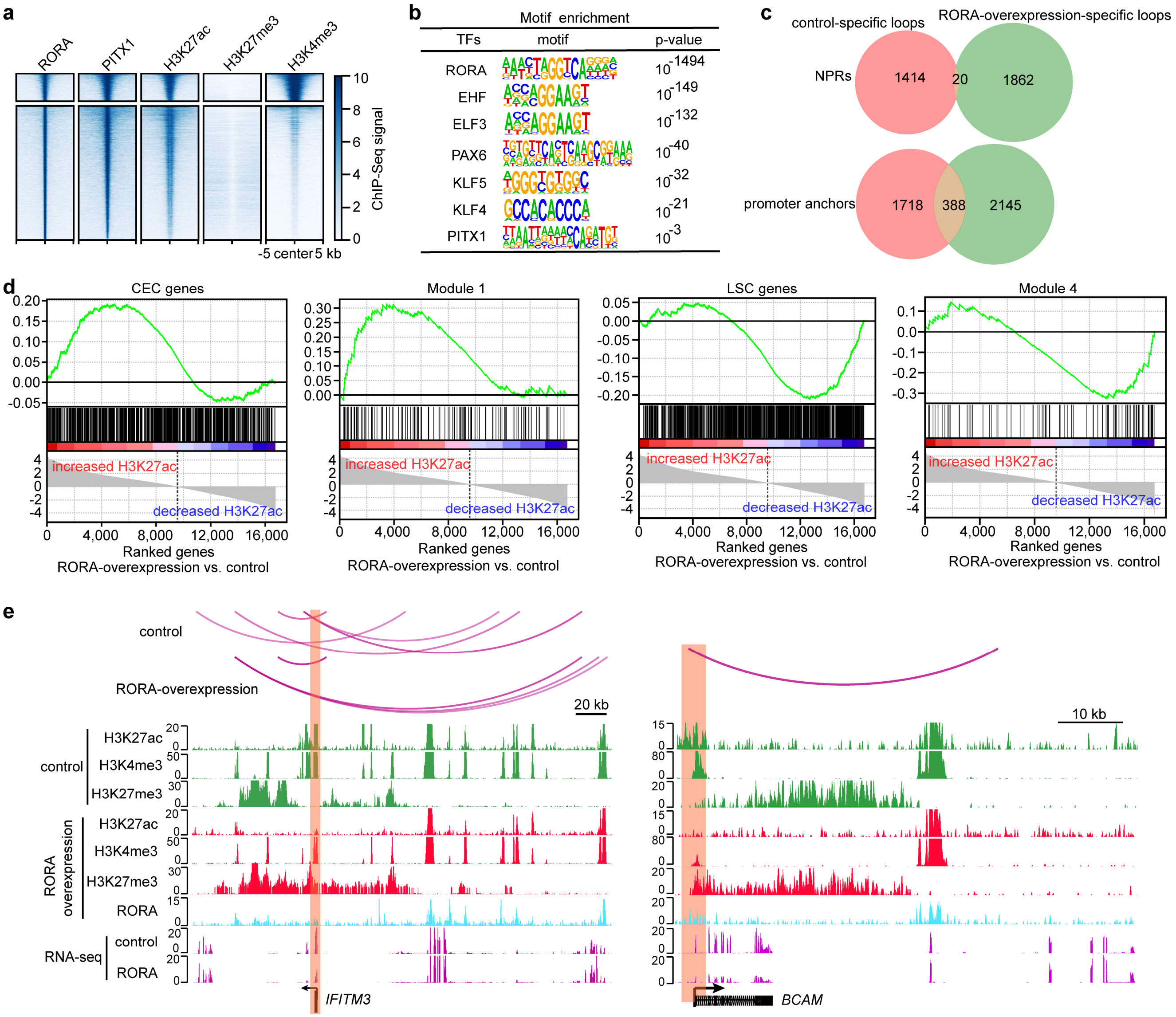

Supplementary Fig. 4 RORA-mediated epigenetic remodeling controls CEC differentiation program. **a** Heatmaps showing the indicated ChIP-seq signals across the center of RORA peaks in *RORA*-overexpressed LSCs. **b** Motif enrichment of RORA binding sites. **c** Vene plots showing the overlapping of promoter or NPR anchors between control specific and *RORA*-overexpression-specific loops. **d** GSEA for the indicated gene sets using H3K27ac ChIP-seq data of *RORA*-overexpression vs. control. **e** Genome browser tracks for promoter-anchored chromatin interactions and the indicated ChIP-seq and RNA-seq signals across the *IFITM3* and *BCAM* loci in control and *RORA*-overexpression LSCs.
