# Supplementary material for: The single-cell transcriptomic atlas and RORA-mediated 3D epigenomic remodeling in driving corneal epithelial differentiation
Source: Nat Commun. 2024 Jan 4;15:256. doi: 10.1038/s41467-023-44471-w (PMC10766623; doi:10.1038/s41467-023-44471-w)
Supplement: Supplementary file 3 — Reporting Summary [file 41467_2023_44471_MOESM3_ESM.pdf]

nature portfolio

Corresponding author(s): Hong Ouyang, Mingsen Li, Jianping Ji

Last updated by author(s): Nov 26, 2023

Reporting Summary

Nature Portfolio wishes to improve the reproducibility of the work that we publish. This form provides structure for consistency and transparency in reporting. For further information on Nature Portfolio policies, see our Editorial Policies and the Editorial Policy Checklist.

Statistics

For all statistical analyses, confirm that the following items are present in the figure legend, table legend, main text, or Methods section.

n/a	Confirmed	
☐	☒	The exact sample size (*n*) for each experimental group/condition, given as a discrete number and unit of measurement
☐	☒	A statement on whether measurements were taken from distinct samples or whether the same sample was measured repeatedly
☐	☒	The statistical test(s) used AND whether they are one- or two-sided<br>*Only common tests should be described solely by name; describe more complex techniques in the Methods section.*
☒	☐	A description of all covariates tested
☐	☒	A description of any assumptions or corrections, such as tests of normality and adjustment for multiple comparisons
☐	☒	A full description of the statistical parameters including central tendency (e.g. means) or other basic estimates (e.g. regression coefficient) AND variation (e.g. standard deviation) or associated estimates of uncertainty (e.g. confidence intervals)
☐	☒	For null hypothesis testing, the test statistic (e.g. *F*, *t*, *r*) with confidence intervals, effect sizes, degrees of freedom and *P* value noted<br>*Give P values as exact values whenever suitable.*
☒	☐	For Bayesian analysis, information on the choice of priors and Markov chain Monte Carlo settings
☒	☐	For hierarchical and complex designs, identification of the appropriate level for tests and full reporting of outcomes
☒	☐	Estimates of effect sizes (e.g. Cohen's *d*, Pearson's *r*), indicating how they were calculated

Our web collection on statistics for biologists contains articles on many of the points above.

Software and code

Policy information about availability of computer code

Data collection	Sequencing data was obtained from Illumina NovaSeq 6000 system.
Data analysis	trimmomatic tool (version 0.36), BWA (version 0.7.17), MACS2 (version 2.1.1), HOMER (v4.9.1), deepTools (v3.0.2), DiffBind package (version 2.8.0), CHiCAGO (version 1.30.0), HiCUP (v0.8.3), Metascape (version 1.0), Picard Markduplicates (version 2.18.16), Integrative Genomics Viewer (version 2.4.13), WashU EpiGenome browser (v48.5.0), STAR software (version 2.6.1a), RSEM (v1.3.0), DESeq2 (version 1.20.0), GSEA (version 4.0.3), edgeR (v3.32.1), R (v4.0.3), CellRanger (v3.1.0), Seurat (v4.0.2), AUCell (v1.12.0), Scanpy (v1.9.2), CellRank (v1.5.1), hdWGCNA (v0.2.18), Dynamo (v1.3.2), SCENIC (v0.11.2), CellOracle (v0.10.12) and GraphPad Prism (v7.0). The code of bioinformatics analysis in this paper is available through GitHub (https://github.com/Mingsenli/corneal-single-cells).

For manuscripts utilizing custom algorithms or software that are central to the research but not yet described in published literature, software must be made available to editors and reviewers. We strongly encourage code deposition in a community repository (e.g. GitHub). See the Nature Portfolio guidelines for submitting code & software for further information.

Data

Policy information about availability of data

All manuscripts must include a data availability statement. This statement should provide the following information, where applicable:

- Accession codes, unique identifiers, or web links for publicly available datasets
- A description of any restrictions on data availability
- For clinical datasets or third party data, please ensure that the statement adheres to our policy

scRNA-seq data used in this paper were downloaded from the Gene Expression Omnibus under the accession number GSE155683. The raw sequence data generated in this paper have been deposited in the Gene Expression Omnibus under the accession number GSE249150. The RcisTarget motif databases are available at https://resources.aertslab.org/cistarget/databases/homo_sapiens/hg38/refseq_r80/mc9nr/gene_based/.

Research involving human participants, their data, or biological material

Policy information about studies with human participants or human data. See also policy information about sex, gender (identity/presentation), and sexual orientation and race, ethnicity and racism.

Reporting on sex and gender	All normal human limbus tissues were obtained as de-identified surgical specimens from eyebank of Zhongshan Ophthalmic Center. All donor information has been de-identified. Therefore, the sex and gender information were not collected. This study does not focus on sex and gender differences either.
Reporting on race, ethnicity, or other socially relevant groupings	The samples used in this paper are from xanthoderm.
Population characteristics	All samples were collected from healthy people.
Recruitment	No patients recruited occurred for this study. Study samples were obtained from eyebank of Zhongshan Ophthalmic Center.
Ethics oversight	The Ethics Committee of Zhongshan Ophthalmic Center of Sun Yat-sen University

Note that full information on the approval of the study protocol must also be provided in the manuscript.

Field-specific reporting

Please select the one below that is the best fit for your research. If you are not sure, read the appropriate sections before making your selection.

[x] Life sciences   [ ] Behavioural & social sciences   [ ] Ecological, evolutionary & environmental sciences

For a reference copy of the document with all sections, see nature.com/documents/nr-reporting-summary-flat.pdf

Life sciences study design

All studies must disclose on these points even when the disclosure is negative.

Sample size	No statistical methods were used to determine sample sizes. Promoter capture Hi-C and the next-generation sequencing experiments were performed in two replicates, which is sufficient to determine reproducible results based on extensive experience and is widely accepted. All the sample sizes of sequencing data were based on standard sequencing analysis practices and were determined to be suitable for statistical analyses. Cellular and molecular biology experiments were performed in triplicate.
Data exclusions	No data were excluded.
Replication	All results were reliably reproduced in multiple independent experiments as indicated in the figure legends.
Randomization	This study does not involve work that requires random allocation because all experiments are performed in one cell type and no comparisons between experimental groups were made.
Blinding	All experiments were objectives. Blinding was not relevant to this study.

Reporting for specific materials, systems and methods

We require information from authors about some types of materials, experimental systems and methods used in many studies. Here, indicate whether each material, system or method listed is relevant to your study. If you are not sure if a list item applies to your research, read the appropriate section before selecting a response.

Materials & experimental systems

n/a	Involved in the study
☐	☒ Antibodies
☒	☐ Eukaryotic cell lines
☒	☐ Palaeontology and archaeology
☒	☐ Animals and other organisms
☒	☐ Clinical data
☒	☐ Dual use research of concern
☒	☐ Plants

Methods

n/a	Involved in the study
☐	☒ ChIP-seq
☒	☐ Flow cytometry
☒	☐ MRI-based neuroimaging

Antibodies

Antibodies used

The antibodies used for immunofluorescence are as follows: anti-KRT3 (Abcam, #ab68260, 1:200), anti-KRT12 (Abcam, #ab124975, 1:200), anti-LGALS3 (Biolegend, #125401, 1:200), anti-S100A2 (Abcam, #ab109494, 1:200), anti-KRT15 (NeoMarkers, #MS-1068-P0, 1:200), anti-IFTIM3 (Proteintech, #11714-1-AP, 1:200), anti-PAX6 (Sigma, #AMAB91372, 1:200), anti-ALDH3A1 (GeneTex, #GTX30042, 1:200), anti-CLU (Proteintech, #12289-1-AP, 1:200), anti-KRT19 (Biolegend, #628502, 1:200), anti-RORA (Immunoway, #YT4166, 1:100), anti-PITX1 (Sigma, #HPA008743, 1:100), Anti-KRT14 (Thermo, #MA511599, 1:200), Anti-KI67 (Cell Signaling Technology, #9129S, 1:200), Anti-TP63 (Cell Signaling Technology, 67825S, 1:200), anti-rabbit IgG (Alexa Fluor 488 Conjugate, CST, 4412S,1:1000), anti-mouse IgG (Alexa Fluor 488 Conjugate, CST, 4408S,1:1000), anti-rabbit IgG (Alexa Fluor 594 Conjugate, CST, 8889S,1:1000), and anti-mouse IgG (Alexa Fluor 594 Conjugate, CST, 8890S,1:1000).
The antibodies used for ChIP-Seq (5 μg/ChIP) are as follows: anti-H3K27ac (Millipore, #07-360), anti-H3K27me3 (Cell Signaling Technology, #9733), anti-H3K4me3 (Cell Signaling Technology, #9751), anti-FLAG (Cell Signaling Technology, #14793).

Validation

Antibodies used in our study were validated as noted by suppliers. Antibody validation information can be found on manufacturers' website.
anti-KRT3 : https://www.abcam.cn/products/primary-antibodies/cytokeratin-3ck-3-antibody-ae5-ab68260.html
anti-KRT12: https://www.abcam.cn/products/primary-antibodies/keratin-12k12-antibody-epr16092-ab124975.html
anti-LGALS3 : https://www.biolegend.com/en-us/products/purified-anti-mouse-human-mac-2-galectin-3-antibody-4935
anti-S100A2: https://www.abcam.cn/products/primary-antibodies/s100-alpha-2s100a2-antibody-epr5392-ab109494.html
anti-KRT15: https://www.bioz.com/search?q=MS%252D1068%252DP0&other=true&exact_search=true&rating_sort=true
anti-IFTIM3: https://www.ptgcn.com/products/IFITM3-Antibody-11714-1-AP.htm
anti-PAX6: https://www.biolegend.com/en-us/products/purified-anti-pax-6-antibody-11511
anti-ALDH3A1: https://www.genetex.cn/Product/Detail/ALDH3A1-antibody/GTX30042
anti-CLU: https://www.ptgcn.com/products/CLU-Antibody-12289-1-AP.htm
anti-KRT19: https://www.biolegend.com/en-us/products/purified-anti-cytokeratin-19-antibody-2983
anti-RORA : http://immunoway.com/Home/Search?keywords=YT4166
anti-PITX1: https://www.sigmaaldrich.cn/CN/zh/product/sigma/hpa008743
anti-H3K27ac: https://www.sigmaaldrich.cn/CN/zh/product/mm/07360
anti-H3K27me3: https://www.cellsignal.cn/products/primary-antibodies/tri-methyl-histone-h3-lys27-c36b11-rabbit-mab/9733
anti-H3K4me3: https://www.cellsignal.cn/products/primary-antibodies/tri-methyl-histone-h3-lys4-c42d8-rabbit-mab/9751
anti-FLAG: https://www.cellsignal.cn/products/primary-antibodies/dykddddk-tag-d6w5b-rabbit-mab-binds-to-same-epitope-as-sigma-aldrich-anti-flag-m2-antibody/14793

Plants

Seed stocks

Report on the source of all seed stocks or other plant material used. If applicable, state the seed stock centre and catalogue number. If plant specimens were collected from the field, describe the collection location, date and sampling procedures.

Novel plant genotypes

Describe the methods by which all novel plant genotypes were produced. This includes those generated by transgenic approaches, gene editing, chemical/radiation-based mutagenesis and hybridization. For transgenic lines, describe the transformation method, the number of independent lines analyzed and the generation upon which experiments were performed. For gene-edited lines, describe the editor used, the endogenous sequence targeted for editing, the targeting guide RNA sequence (if applicable) and how the editor was applied.

Authentication

Describe any authentication procedures for each seed stock used or novel genotype generated. Describe any experiments used to assess the effect of a mutation and, where applicable, how potential secondary effects (e.g. second site T-DNA insertions, mosiacism, off-target gene editing) were examined.

ChIP-seq

Data deposition

☒ Confirm that both raw and final processed data have been deposited in a public database such as GEO.

☒ Confirm that you have deposited or provided access to graph files (e.g. BED files) for the called peaks.

Data access links
May remain private before publication.

https://ngdc.cncb.ac.cn/gsa-human

Files in database submission

control_H3K27AC_1
control_H3K27AC_2
control_H3K27me3_1
control_H3K27me3_2
control_H3K4me3_1
control_H3K4me3_2
RORA_H3K27ac_1
RORA_H3K27ac_2
RORA_H3K27me3_1
RORA_H3K27me3_2
RORA_H3K4me3_1
RORA_H3K4me3_2
RORA_flag_1
RORA_flag_2
control-input
RORA-OE-input

Genome browser session
(e.g. UCSC)

Not applicable

Methodology

Replicates

Two independent experimental replications were performed.

Sequencing depth

8Gb paired-end data were generated

Antibodies

anti-H3K27ac (Millipore, #07-360), anti-H3K27me3 (Cell Signaling Technology, #9733), anti-H3K4me3 (Cell Signaling Technology, #9751), anti-Flag (Cell Signaling Technology, #14793).

Peak calling parameters

-f BAMPE -B --SPMR -q 0.01 --call-summits --fix-bimodal --seed 11521 --extsize 200

Data quality

FastQC(v0.11.8) was used to assess the data quality

Software

trimmomatic tool (version 0.36), BWA (version 0.7.17), MACS2 (version 2.1.1), DiffBind package (version 2.8.0), Integrative Genomics Viewer (version 2.4.13), and deepTools (v3.0.2)
